# Digital hearts: advancing cardiovascular health through data-science-driven clinical translation

**DOI:** 10.1016/j.mmr.2026.100074

**Published:** 2026-10-17

**Authors:** Yu-Hong Hu, Xi-Jia Shao, Jiang-Ping Song

**Affiliations:** Department of Cardiac Surgery, Fuwai Hospital, National Center for Cardiovascular Diseases, Chinese Academy of Medical Sciences and Peking Union Medical College, Beijing 100037, China; State Key Laboratory of Cardiovascular Disease, Fuwai Hospital, National Center for Cardiovascular Diseases, Chinese Academy of Medical Sciences and Peking Union Medical College, Beijing 100037, China; Clinical Research Institute, Chinese Academy of Medical Sciences, Faculty of Clinical Medicine, Peking Union Medical College, Beijing 100730, China; Department of Cardiac Surgery, Fuwai Hospital, National Center for Cardiovascular Diseases, Chinese Academy of Medical Sciences and Peking Union Medical College, Beijing 100037, China; State Key Laboratory of Cardiovascular Disease, Fuwai Hospital, National Center for Cardiovascular Diseases, Chinese Academy of Medical Sciences and Peking Union Medical College, Beijing 100037, China; Clinical Research Institute, Chinese Academy of Medical Sciences, Faculty of Clinical Medicine, Peking Union Medical College, Beijing 100730, China; Beijing Key Laboratory for Organ Xenotransplantation, Fuwai Hospital, National Center for Cardiovascular Diseases, Chinese Academy of Medical Sciences and Peking Union Medical College, Beijing 100037, China; Shenzhen State Key Laboratory of Cardiovascular Disease, Fuwai Hospital, Chinese Academy of Medical Sciences, Shenzhen 518057, Guangdong, China

**Keywords:** Digital heart, Digital twins, Artificial intelligence, Clinical translation

Dear Editor,

Digital models differ from biological systems because they can be duplicated, repeatedly tested, and iteratively updated at relatively low additional cost. Patient-specific computational heart models are increasingly being investigated as tools that use clinical data to enable individualized in silico simulations [1]. Imaging-based patient-specific models have been used to identify arrhythmogenic substrates and propose ablation targets in ischemic ventricular tachycardia [2]. A recent 10-patient feasibility study also prospectively applied digital twin guidance during ablation, although these findings remain preliminary and require larger comparative studies [3].

A principal advantage of digital heart modeling is controlled counterfactual testing (Fig. 1**a**). Clinical cardiology is constrained by ethical limitations and the irreversibility of many interventions, which often preclude direct experimentation on pivotal mechanistic questions. Digital heart models enable individual factors to be varied in silico while other relevant factors are kept unchanged. This approach can help compare ablation strategies and assess how differences in scar distribution and geometry affect arrhythmia susceptibility [1], [2]. Such controlled in silico testing may support clinical decision-making under uncertainty by linking predicted outcomes to underlying physiological mechanisms.

**Fig. 1:**
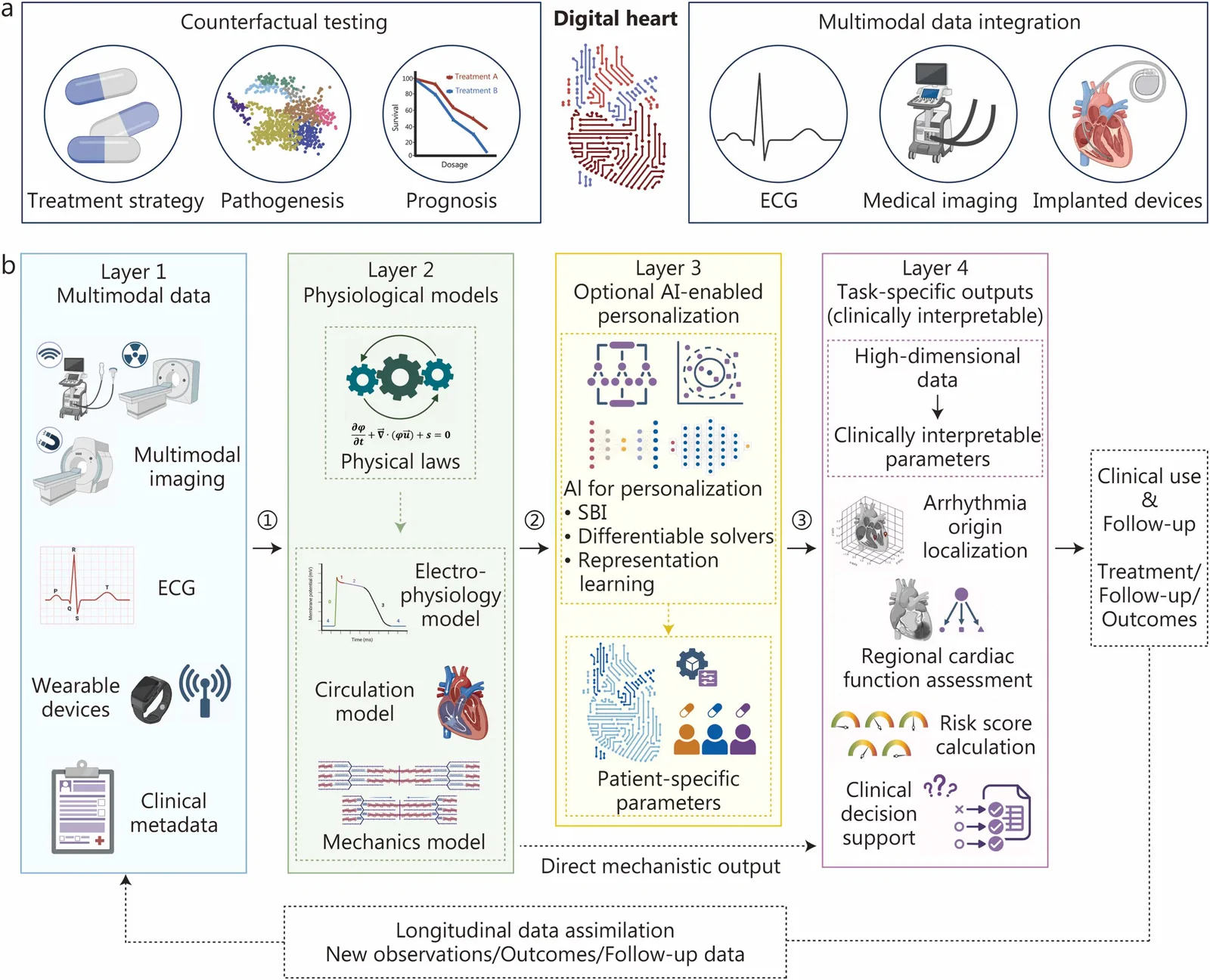
**Conceptual framework of the digital heart. a** Two principal capabilities of digital hearts: counterfactual testing and integration of multimodal data. **b** Four-layer architecture showing information flow from multimodal patient data through physiological models and optional AI-enabled personalization to task-specific, clinically interpretable outputs and subsequent clinical use. Solid arrows indicate forward information flow, whereas the direct mechanistic pathway illustrates that AI-enabled personalization is optional. The dashed feedback loop represents longitudinal assimilation of new observations, treatment responses, and follow-up outcomes into the patient representation. Mechanistic and AI-enabled components are visually separated to emphasize their distinct roles within the framework. AI. Artificial intelligence; ECG. Electrocardiogram; SBI. Simulation-based inference.

The second potential advantage is the integration of multimodal patient data within a computational framework (Fig. 1**a**). Modern clinical cardiology routinely generates multimodal patient data that provide complementary information about cardiac structure, function, and clinical status. Imaging can characterize cardiac anatomy, tissue composition, and fibrosis. Electrocardiography captures body-surface electrical activity, whereas invasive mapping provides detailed information about intracardiac electrical activation. Longitudinal data from comorbidities, implanted devices, and wearable sensors provide additional information about changes in physiological state over time. In hybrid digital heart models, mechanistic models can provide a common physiological framework for integrating multimodal patient data. These data can be interpreted according to known physiological principles to generate outputs such as virtual electrocardiograms, pressure-volume loops, regional stress or strain estimates, activation maps, and arrhythmogenic substrate maps [1], [2].

Here, digital heart refers to a broad range of computational representations of cardiac structure, function, or disease, spanning population-based, subgroup-specific, and patient-specific models developed using mechanistic, data-driven, or hybrid approaches. Because “digital twin” is defined inconsistently across the literature, we do not propose a single rigid taxonomy. We use “clinical digital twin” to describe a patient-specific digital heart model that is integrated into a defined clinical workflow and governed throughout its lifecycle. Repeated or event-triggered data assimilation is considered an advanced feature rather than a requirement for all clinical digital twins. Accordingly, we use “digital heart” throughout when referring to the broader class of models, and “clinical digital twin” only when referring specifically to this clinically integrated subset. This distinction reflects differences in model maturity and clinical integration. However, the terminology used for a model does not by itself determine the level of evidence or regulatory requirements that apply to it. Existing digital heart models range from one-time patient-specific ablation-planning models [2] to early prospectively deployed systems [3]. More generally, digital heart models may vary along several descriptive dimensions. These include the clinical context of use, modeled substrate, degree of patient specificity, data-assimilation strategy, model composition (mechanistic, data-driven, or hybrid), integration into clinical workflow, and lifecycle governance. These dimensions describe model scope and maturity rather than its functional architecture. Accordingly, the rigor of evaluation should depend on the model’s intended clinical use, the potential harm if it contributes to an incorrect clinical decision, and how much clinicians rely on its output. Digital heart models that are not continuously updated may still support procedural planning, help avoid unnecessary ablation lesions, and help determine which of several possible mechanisms best explains the observed findings. Translational progress should therefore be judged by whether a model provides meaningful improvement over existing approaches for a predefined clinical task, rather than by whether it includes all features of a fully developed digital twin.

In our framework, a digital heart model is organized into 4 functional layers (Fig. 1**b**). Layer 1 contains multimodal patient data, including imaging, electrical recordings, wearable signals, and clinically relevant metadata. Layer 2 contains models of electrophysiology, cardiac mechanics, and circulation that represent key physical and physiological relationships. Layer 3 comprises optional artificial intelligence (AI)-enabled personalization, including simulation-based inference, differentiable solvers, and representation learning for estimating patient-specific parameters and their uncertainty. Pretrained cardiovascular foundation models may also contribute to this layer by supporting image segmentation, phenotype extraction, data harmonization, or parameter initialization [4], but they do not by themselves establish mechanistic validity, patient specificity, or clinical value. Layer 4 contains task-specific modules that translate simulated physiological states into clinically interpretable outputs. Here, clinically interpretable outputs refer to model outputs that can be directly related to a predefined clinical question, such as physiological variables, spatial maps, or simulation-derived endpoints. Examples include activation or arrhythmogenic substrate maps, pressure-volume relationships, regional assessments of impaired myocardial strain, and individualized risk estimates with uncertainty ranges. The above content describes the functional organization of a digital heart model. The following considerations instead concern how such models should be designed, personalized, extended, and evaluated for a specific clinical task.

The development process should begin with a clearly defined clinical context of use. The model is then personalized using patient data relevant to that clinical task. Patient- or subgroup-specific parameters are estimated and calibrated against observed data, after which the model is tested against independent measurements before counterfactual simulations are performed. The resulting outputs are translated into task-specific predictions with explicit uncertainty.

Patient specificity can vary in degree. A model may be personalized anatomically, functionally, or both. A digital heart does not necessarily require a multiscale three-dimensional model or an AI component. Population reference models may be stratified or adapted for subgroups defined by genotype, biological sex, age, disease phenotype, or baseline structural and functional status, and subsequently personalized to individual patients. Such models may support research and training as well as context-specific clinical applications, including virtual comparison of ablation strategies, device settings, or pharmacological interventions. Repeated assimilation of new patient data can enable a patient-specific model to evolve toward a longitudinal clinical digital twin. The digital heart should therefore not be regarded as a single comprehensive model, but as a context-dependent computational representation whose scope and level of personalization depend on the intended question.

The physiological scope of a digital heart model should be determined by the clinical question. In some settings, cardiac function and treatment response are influenced by extracardiac systems, which should be incorporated when they materially affect the simulated outcome. For example, autonomic regulation can modulate heart rate, conduction, contractility, and arrhythmia susceptibility, whereas coupling cardiac models with the systemic and pulmonary circulations can provide more realistic preload and afterload conditions and capture bidirectional interactions between ventricular function and vascular haemodynamics [5]. However, these extensions are not necessary for all digital heart models. The degree of extracardiac or multiorgan integration should depend on the intended context of use and the relevance of these interactions to the clinical question. Furthermore, experimental cellular and tissue models could enable digital heart models to incorporate multiple biological scales. Induced pluripotent stem cell-derived cardiomyocytes (iPSC-CMs) generated from patient cells, engineered cardiac tissues, and cardiac organoids can provide data on electrophysiology, contractility, molecular profiles, and pharmacological responses. These data can be used to refine computational models at the cellular and tissue levels and to test whether model predictions are consistent with experimental results under controlled conditions. Computational models calibrated using experimental data from iPSC-CMs have demonstrated the feasibility of linking observed cellular phenotypes to individualized electrophysiological models and drug-response predictions [6]. More broadly, integration of these cell and tissue models with multi-omics data could connect gene expression patterns and cellular phenotypes to tissue properties and clarify how molecular changes influence cardiac tissue and whole-heart function.

As digital heart models move from research toward clinical use, establishing their credibility becomes essential. Consistent with ASME V&V 40 and the U.S. Food and Drug Administration framework for computational modeling and simulation, the required level of model credibility should be risk-informed and commensurate with the model’s influence on a clinical decision and the consequences of an incorrect decision [7], [8]. Accordingly, model evaluation should combine appropriate technical verification and validation against independent data with external multicenter validation, prospective evaluation, calibration, reproducibility assessment, and uncertainty quantification, as appropriate to the intended context of use. For regulatory applications, benchmarking against fit-for-purpose reference standards may also be required before the model is used to support clinical decisions that could substantially affect patient care. Continuously updated systems additionally require traceable records of data sources and processing, predefined update rules, and version histories. For AI-enabled components, ongoing oversight should include post-deployment performance monitoring, model modification, and management of dataset drift [9]. For hybrid physics-AI systems, credibility assessment of the mechanistic component should be complemented by separate evaluation of AI data quality, domain shift, subgroup performance, and post-deployment behavior [9].

Deployment requirements will also vary across healthcare environments. In resource-constrained settings such as field hospitals, task-specific models could use limited locally acquired imaging, electrocardiographic, or hemodynamic data to support remote computational consultation, provided that their performance remains robust to missing modalities and heterogeneous acquisition. In integrated health systems such as Veterans Affairs hospitals, longitudinal clinical, imaging, device, and wearable data could instead support repeated model updating for chronic disease surveillance, treatment planning, and follow-up. Thus, model complexity and data requirements should be adapted to the deployment setting and intended clinical task.

Over the next 5 years, research should prioritize clinical indications in which counterfactual simulation can provide mechanistic insight, including ventricular tachycardia ablation, phenotyping of heart failure with preserved ejection fraction (HFpEF), and risk stratification in inflammatory cardiomyopathy. Shared benchmarks, external validation, and prospective trials will be essential for establishing reproducible performance and clinical value. HFpEF may be particularly suited to digital heart modeling because similar clinical phenotypes can arise from heterogeneous combinations of impaired relaxation, ventricular and vascular stiffness, altered loading conditions, chronotropic dysfunction, and non-cardiac comorbidities that are difficult to distinguish using a single data modality [10]. The objective is not to replace clinical judgment but to support it through reproducible, physiologically grounded simulations that clinicians can interpret and critically evaluate.

## Abbreviations

AI: Artificial intelligence
iPSC-CMs: Induced pluripotent stem cell-derived cardiomyocytes
HFpEF: Heart failure with preserved ejection fraction

## Ethics approval and consent to participate

Not applicable.

## Authors’ contributions

YHH conceived the concept, designed the framework, and drafted the manuscript. XJS prepared the figure and reviewed and revised the manuscript. JPS conceived the concept and reviewed and revised the manuscript. All authors read and approved the final manuscript.

## Consent for publication

Not applicable.

## Funding

This work was supported by the National Natural Science Fund for Distinguished Young Scholars of China (82125004), the Frontier Biotechnology Key Project of the National Key R&D Program of the Ministry of Science and Technology of China (2023YFC3404300), and the Special Project of the Ministry of Science and Technology of China supported by the State Key Laboratory of Cardiovascular Disease, Fuwai Hospital, Chinese Academy of Medical Sciences (2024GZZD-03).

## Competing interest

The authors declare that they have no competing interests.

## Data Availability

Not applicable.
